# Niclosamide and its derivative DK‐520 inhibit RANKL‐induced osteoclastogenesis

**DOI:** 10.1002/2211-5463.12921

**Published:** 2020-07-22

**Authors:** Yurui Jiao, Chenglong Chen, Xijian Hu, Xu Feng, Zhenqi Shi, Jie Cao, Qing Li, Yikun Zhu

**Affiliations:** ^1^ Department of Endocrinology The Second Hospital of Shanxi Medical University Taiyuan China; ^2^ Musculoskeletal Tumor Center Peking University People’s Hospital Beijing China; ^3^ Department of Orthopedics The Second Hospital of Shanxi Medical University Taiyuan China; ^4^ Department of Pathology University of Alabama at Birmingham AL USA

**Keywords:** differentiation, DK‐520, Niclosamide, osteoclast, RANKL

## Abstract

Niclosamide is a potent inhibitor of osteoclastogenesis and bone remodeling. DK‐520 is an acyl derivative of Niclosamide and significantly increased both the plasma concentration and the duration of exposure of Niclosamide when dosed orally. However, at present the effect of DK‐520 on osteoclastogenesis has not been reported. Here, we investigated whether DK‐520 can regulate receptor activator of nuclear factor‐κB ligand (RANKL)‐induced osteoclastogenesis of bone marrow macrophages (BMMs) *in vitro*. Following induction of BMMs with RANKL for three days, we detected differentiated osteoclasts with typical morphology and high levels of tartrate‐resistant acid phosphatase (TRAP), RANKL, and cathepsin K (CTSK) expression. Treatment with either Niclosamide or DK‐520 did not affect the viability of osteoclast precursors (OCPs), but significantly inhibited RANKL‐induced transdifferentiation of macrophages into OCPs, particularly in the early stage of osteoclastogenesis. Both Niclosamide and DK‐520 significantly decreased the relative levels of transcription factor PU.1 mRNA transcripts and dendritic cell‐specific transmembrane protein (DC‐STAMP), but not v‐ATPasev_0_d_2_ protein expression in OCPs. In addition, the inhibitory effect of DK‐520 on osteoclastogenesis is realized through impairment of the NF‐kB (nuclear factor‐κB) and MAPK (mitogen‐activated protein kinase) signaling pathways. These results demonstrate that DK‐520, like Niclosamide, effectively inhibits the early stage of osteoclastogenesis. The findings presented here, together with its increased oral plasma concentrations and bioavailability, suggest that DK‐520 may be a promising drug candidate for treatment of osteoclast‐related diseases.

## Graphical abstract

DK‐520 is an acyl derivative of Niclosamide that exhibits a significant increase in both the plasma concentration and bioavailability. Like Niclosamide, DK‐520 effectively inhibits the early stages of RANKL‐induced osteoclastogenesis and may be a promising drug candidate for treatment of osteoclast‐related diseases.

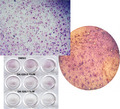

**Abbreviations**
BMMs: bone marrow macrophages
c‐fms: colony‐stimulating factor 1 receptor
CTSK: cathepsin K
DC‐STAMP: dendritic cell‐specific transmembrane proteins
ERK: extracellular signal‐regulated kinase
FBS: fetal bovine serum
IκB: inhibitor of NF‐κB
JNK: Janus N‐terminal kinase
MAPK: mitogen‐activated protein kinase
M‐CSF: macrophage colony‐stimulating factor
NFATc1: nuclear factor of activated T cells c1
NF‐κB: nuclear factor‐κB
OCPs: osteoclast precursors
RANK: receptor activator of nuclear factor‐κβ
RANKL: receptor activator of nuclear factor‐κβ ligand
SD: standard deviation
SERMs: selective estrogen receptor modulators
TRAP: tartrate‐resistant acid phosphatase
α‐MEM: alpha‐minimum essential medium

Osteoporosis is a common disease that affects many millions of peoples, particularly older men and postmenopausal women [1]. Pathologically, osteoporosis is characterized by reduced bone mass and altered bone microstructure, as well as increased risk for bone fractures. Osteoporotic fractures and their complications can cause disability and death in older patients. In addition, medical care for osteoporosis and osteoporotic fractures requires numerous resources and financial costs, which can be a socioeconomic burden for many families globally [2]. Osteoporosis is attributed to metabolic imbalance between bone formation by osteoblasts and bone resorption by osteoclasts [3].

Osteoclasts are multinucleated giant cells that are mainly located around vascular channels in the surface of the bone and are derived from the fusion of multiple monocytes/macrophages [4]. During the process of osteoclast differentiation and maturation, receptor activator of nuclear factor κβ ligand (RANKL) on osteoblasts and stromal cells, and macrophage colony‐stimulating factor (M‐CSF) activate nuclear factor‐kB (NF‐kB) and MAPK (mitogen‐activated protein kinase) signaling to induce expression of NFATc1 (nuclear factor of activated T cells c1) and other factors, causing macrophage activation and differentiation into osteoclast precursor cells (OCPs). Subsequently, OCPs fuse and further maturate into osteoclasts [5]. Hence, inhibition of osteoclastogenesis is valuable for controlling osteoporosis. Bisphosphonates, estrogen, or selective estrogen receptor modulators (SERMs) and calcitonin, which inhibit the different stages of osteoclastogenesis, have been used to treat osteoporosis [3]. However, long‐term treatment with these drugs may cause severe side effects, such as osteonecrosis, thromboembolism, atypical femoral fractures, and increased risk of endometrial cancer, breast cancer, and cardiovascular disease [6, 7, 8, 9]. In addition, experimental studies have revealed that inhibitors of cathepsin K (CTSK), c‐Src, and αVβ3 integrin receptor can inhibit the differentiation and resorption function of osteoclasts [10, 11, 12], but these drugs have not been used in clinical practice. Thus, discovery of new and safe drugs that inhibit osteoclastogenesis will be of significance for managing patients with osteoporosis and osteoporotic fractures.

Niclosamide, an anthelmintic drug, has been used to treat intestinal tapeworm infection since 1982 [13]. DK‐520 is an acyl derivative of Niclosamide and has the unique property that it increased both the plasma concentration and the duration of exposure of Niclosamide *in vivo* with no observable adverse effects [14, 15, 16]. Previous studies have shown that Niclosamide can suppress osteoclastogenesis and bone remodeling, although its precise mechanisms have not been clarified [17, 18]. Furthermore, it is unclear whether and how treatment with DK‐520, like Niclosamide, can inhibit the differentiation of mouse bone marrow macrophages (BMMs) into osteoclasts.

In the present study, we utilized a mouse BMM model to investigate the mechanisms by which DK‐520 and Niclosamide regulate osteoclastogenesis. Our data indicate that DK‐520, like Niclosamide, inhibits early differentiation of BMMs into osteoclasts *in vitro*. Therefore, DK‐520 may be a promising anti‐osteoporosis drug.

## Materials and Methods

### Special chemicals and reagents

Niclosamide and its derivative DK‐520 were provided by Chen in the Department of Medicine, Duke University Medical Center, Durham, USA; alpha‐minimum essential medium (α‐MEM) and fetal bovine serum (FBS) were from Gibco (Thermo Fisher Scientific, Waltham, MA, USA); soluble recombinant M‐CSF and RANKL were from PeproTech (London, UK); and antibodies against DC‐STAMP and ATPasev_0_d_2_ were from Thermo Fisher Scientific and Abcam (Cambridge, UK). The following primary antibodies and secondary antibodies were purchased from Cell Signaling Technology, Inc.: primary antibodies against NFATc1 and c‐Fos, and secondary antibodies against IκBα, phosphor‐IκBα, p44/42ERK, phospho‐44/42ERK, JNK, phospho‐JNK, p38, and phospho‐p38.

### Animals

Female and male C57BL/6 mice (4–6 weeks of age) were obtained and maintained in the specific pathogen‐free animal facility of Shanxi Medical University (Taiyuan, China). The experimental procedures were conducted according to the Guide for the Care and Use of Laboratory Animals and approved by the specific committee of Shanxi Medical University.

### Induction of osteoclasts

BMMs were isolated from the hind limb long bones of mice. BMMs (5 × 10^4^ cells/well) were treated with or without M‐CSF (44 ng·mL^−1^) and RANKL (100 ng·mL^−1^) in 10% FBS α‐MEM in 48‐well plates in the presence or absence of Niclosamide or DK‐520 at 37℃ and 5% CO_2_ for 3–4 days. The cells were exposed to fresh medium every 2 days. The differentiated cells were characterized for tartrate‐resistant acid phosphatase (TRAP) activity using a TRAP enzymatic staining kit (Sigma‐Aldrich, St. Louis, MO, USA). TRAP^+^ cells with multiple nuclei were counted as multinucleated osteoclasts in a blinded manner. In addition, BMMs were treated in triplicate with M‐CSF and RANKL for 3–4 days and the cells were treated with vehicle DMSO or each compound at 0.75 or 1.5 µm daily. TRAP expression was enzymatically stained, and the relative levels of PU.1 mRNA transcripts and DC‐STAMP and v‐ATPase_0_d_2_ protein expression were determined daily using quantitative Real‐time‐PCR (qRT‐PCR) and western blot, respectively, for the first 2 days.

### CCK‐8 cell viability assay

BMMs (7 × 10^3^ cells/well) were treated in triplicate with vehicle DMSO or varying concentrations of Niclosamide or DK‐520 (0.75, 1.0, 1.25, 1.5, 1.75, 2.0, and 2.25 μm) in the presence of M‐CSF (44 ng·mL^−1^) in 96‐well plates for 24 h to determine OCP viability. BMMs (8 × 10^3^ cells/well) were then cultured with M‐CSF (44 ng·mL^−1^) and RANKL (100 ng·mL^−1^) for 3 days to induce osteoclast maturation. The cells were treated with DMSO or different concentrations of Niclosamide or DK‐520 in the presence of M‐CSF (44 ng·mL^−1^) and RANKL (100 ng·mL^−1^) for 24 h. Cell viability was measured using the CCK‐8 assay kit (Dojindo Molecular Technologies, Tokyo, Japan).

### In vitro bone resorption assays

BMMs (1 × 10^5^ cells/well) were seeded on bovine cortical bone slices plated in 24‐well tissue culture plates, and then cultured in the presence of M‐CSF (44 ng·mL^−1^) and RANKL (100 ng·mL^−1^) with or without DK‐520 for up to 5 days to promote osteoclastogenesis and bone resorption. Then, bone slices were harvested and cells were removed from bone slices with sodium hypochlorite solution, and bone slices were dyed with 0.5% Toluidine blue (Fisher Science Education). Images were obtained by microscope, and the data were quantified by measuring the number of resorbed pits in five random resorption areas of one slice.

### Quantitative Real‐time‐PCR

Following osteoclast induction, we examined the impact of each compound on TRAP and CTSK mRNA transcripts using qRT‐PCR. Similarly, we detected the PU.1 mRNA transcript after treatment with each compound daily. Briefly, total RNA was extracted from osteoclasts using a miRNeasy Mini Kit (Qiagen, Germantown, MD, USA) and reverse‐transcribed into cDNA using the PrimeScript RT Master Mix Kit (Takara, Shiga, Japan), followed by qRT‐PCR with the SYBR Premix Ex Taq™ (Takara) and specific primers. The primer sequences were forward 5′AGCAGCCAAGGAGGACTACGTT3′ and reverse 5′TCGTTGATGTCGCACAGAGG3′ for *TRAP*; forward 5′CCTACGCACAAGGCGAAGATGC3′ and reverse 5′CTGAGACCACGATGATGTCGCC3′ for *RANK*; forward 5′CAGCAGAACGGAGGCATTGA3′ and reverse 5′CCTTTGCCGTGGCGTTATAC‐3′ for *CtsK*; forward 5′GGAGCTCAGCTGGATGTTACAGG3′ and reverse 5′GTAGTAGTCATGCATTGGACGTTGG3′ for *PU.1*; and forward 5′TTGTTACCAACTGGGACGACATGG3′ and reverse 5′GATCTTGATCTTCATGGTGCTAGG3′ for *β‐actin*. The data were analyzed using the 2^−ΔΔCt^ method.

### Western blot analysis

Following OCP induction on each day, we examined the impact of each compound on DC‐STAMP and ATPase_0_d_2_ expression daily using western blot analysis. Briefly, the cells were harvested and lysed. Their cytoplasmic and nuclear proteins were extracted using the special reagents (Thermo Fisher Scientific). Subsequently, we characterized these lysates using western blot analysis with anti‐DC‐STAMP and anti‐ATPasev_0_d_2_ primary antibodies and horseradish peroxidase‐conjugated secondary antibodies. In different time periods with or without impacting DK‐520, cells were washed twice with PBS and then lysed in the lysis buffer, supplemented with protease inhibitor (Sigma‐Aldrich) and phosphatase inhibitor cocktails 1 (Sigma‐Aldrich) and 2 (Sigma‐Aldrich). Cytoplasmic and nuclear extracts were prepared using the NE‐PER Nuclear and Cytoplasmic Extraction Reagents (Catalog Thermo Scientific). Lysates were then processed by western blot analysis as previously described [19]. Membranes were incubated with the primary antibodies and horseradish peroxidase‐conjugated secondary antibodies. An ECL detection assay was performed using a SuperSignal West Dura Kit from Pierce (Rockford, IL, USA). The data were analyzed using ImageJ software (National Institute of Health, Bethesda, MD, USA).

### Statistical analysis

Data are expressed as mean ± standard deviation (SD). We statistically analyzed the difference among groups using the Student's *t*‐test and the Statistical Package for the Social Sciences software (SPSS, version 21.0, Chicago, IL, USA). Statistical significance was accepted when the *P*‐value < 0.05.

## Results

### Characterization of induced osteoclasts

To induce osteoclasts, BBMs were isolated and stimulated with or without M‐CSF and RANKL for 3 days. The expression of TRAP was determined using enzymatic staining. As shown in Fig. 2A, there were many TRAP^+^ osteoclasts with several vacuoles and multiple nuclei, and the TRAP^+^ cells displayed a large cytoplasm and round, oval, or irregular body type with uniform red or purple particles. The qRT‐PCR results showed high levels of TRAP, RANK, and CTSK mRNA transcripts in these cells, but not in the control macrophages (Fig. 2B). Together, these data indicate that treatment with M‐CSF and RANKL induces transdifferentiation of BMMs into mature osteoclasts *in vitro*.

### The safe concentrations of Niclosamide and DK‐520

**Fig. 1:**
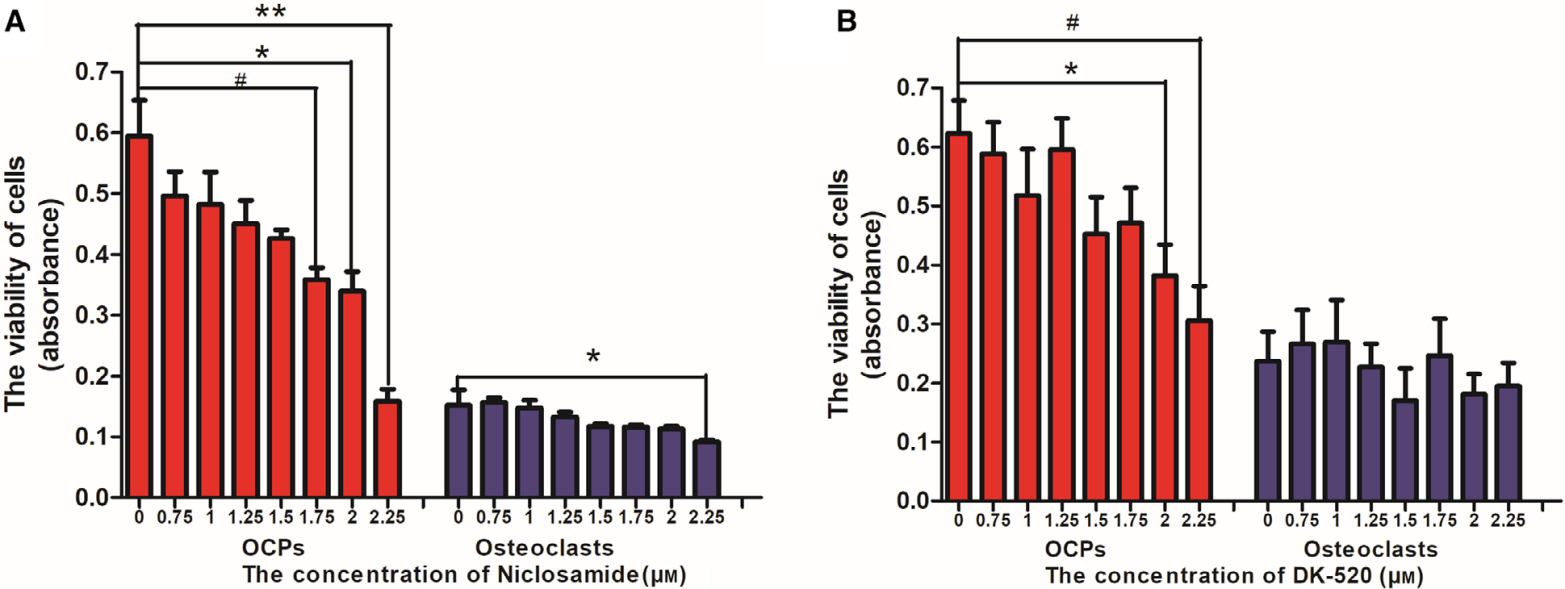
The effects of DK‐520 and Niclosamide on osteoclast viability. BMMs were treated in triplicate with vehicle DMSO or the indicated concentrations of Niclosamide or DK‐520 in the presence of M‐CSF (44 ng·mL^−1^) for 24 h to assay OCP viability. Following induction of osteoclast differentiation for 3 days, the differentiated cells were tested. Cell viability was determined using the CCK‐8 assay. (A) Effects of Niclosamide on OCP viability and mature osteoclasts. (B) The effects of DK‐520 on OCP viability and mature osteoclasts. Data are expressed as the mean ± SD of each group from three separate experiments (*n* = 3). The data were statistically analyzed by Student’s *t*‐test. ^*^ *P* < 0.05, ^**^ *P* < 0.001, ^#^ *P* < 0.01.

First, we investigated the impact of both Niclosamide and DK‐520 on the viability of OCPs and osteoclasts *in vitro*. BMMs were treated with vehicle or different concentrations of either Niclosamide or DK‐520 in the presence of M‐CFS for 24 h, and their viability was determined using the CCK‐8 assay. Treatment with Niclosamide at < 1.75 μm or DK‐520 at < 2.0 μm did not affect the viability of OCPs in our experimental conditions (Fig. 1). Furthermore, following induction of osteoclast maturation, treatment with Niclosamide at 2.25 μm or DK‐520 at dose did not alter the viability of osteoclasts. These data provided a safe dose of each compound for further experiments.

### Both Niclosamide and DK‐520 inhibit RANKL‐induced osteoclastogenesis and bone resorption in vitro

**Fig. 2:**
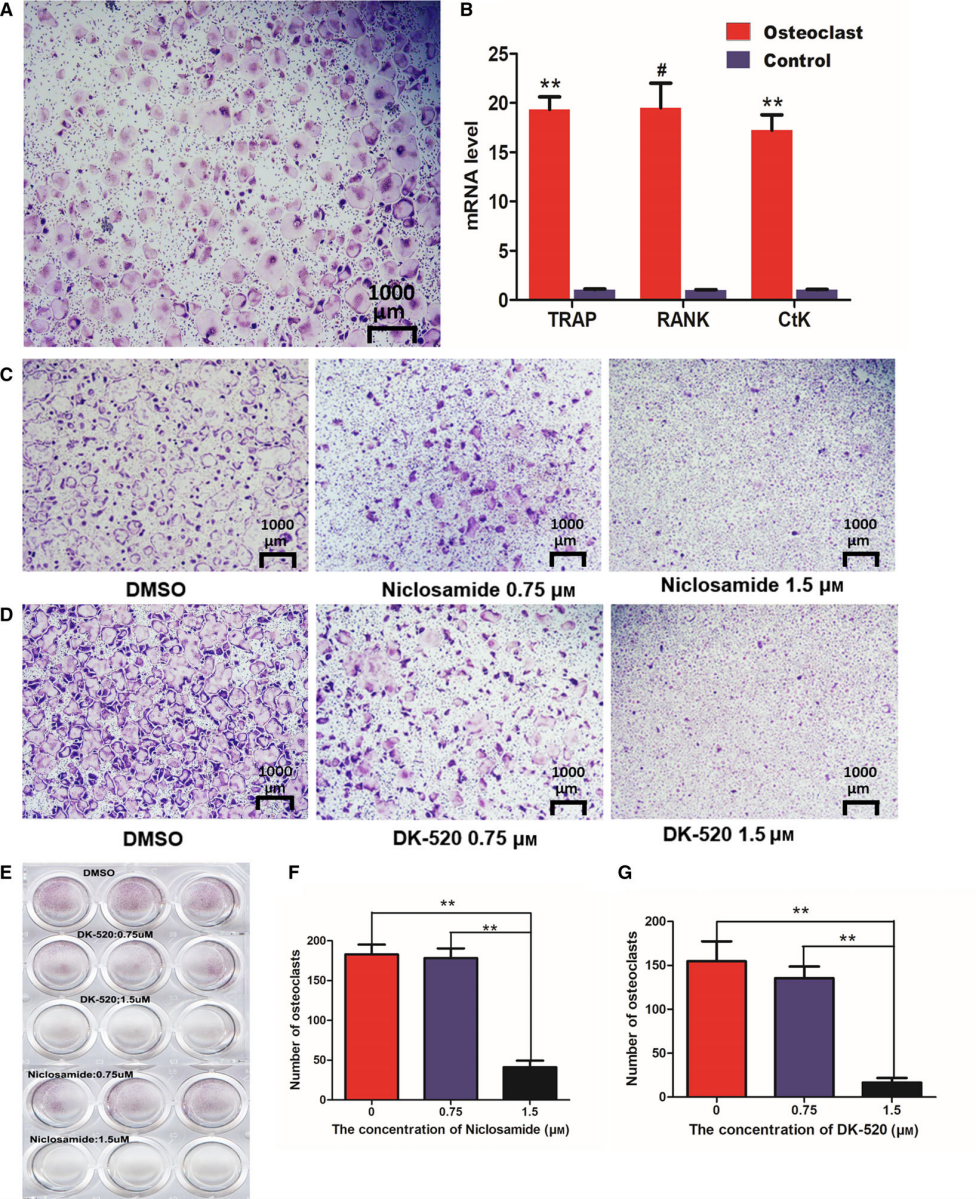
Characterization of differentiated osteoclasts, and both DK‐520 and Niclosamide inhibit osteoclast differentiation. BMMs were treated with M‐CSF and RANKL in the presence or absence of the indicated concentrations of Niclosamide or DK‐520 for 3–4 days. The cells were enzymatically assayed for TRAP activity, and the relative levels of TRAP, RANK, and CTSK mRNA transcripts were determined using qRT‐PCR. The TRAP^+^ cells with multiple nuclei were counted. Data are representative images (magnification × 40) or expressed as the mean ± SD of each group from three separate experiments. (A) Representative image of TRAP staining (magnification × 40). (B) Levels of TRAP, RANK, and CTSK mRNA transcripts. Data are representative images or expressed as the mean ± SD of each group from three separate experiments (*n* = 3). (C) Cell morphology and TRAP staining after 24 h of Niclosamide intervention with different concentrations under an inverted phase‐contrast microscope. (D) Cell morphology and TRAP staining after 24 h of DK‐520 intervention with different concentrations under an inverted phase‐contrast microscope. (E) TRAP staining of cells in the culture plate. (F) Cell number after 24 h of Niclosamide intervention with different concentrations under an inverted phase‐contrast microscope. Data are expressed as the mean ± SEM of each group from three separate experiments (*n* = 4) (G) Cell number after 24h of DK‐520 intervention with different concentrations under an inverted phase‐contrast microscope. Scale bar: 1000 μm. ^**^ *P* < 0.001, ^#^ *P* < 0.01. Data are expressed as the mean ± SEM of each group from three separate experiments (*n* = 3). The data were statistically analyzed by Student’s *t*‐test.

**Fig. 3:**
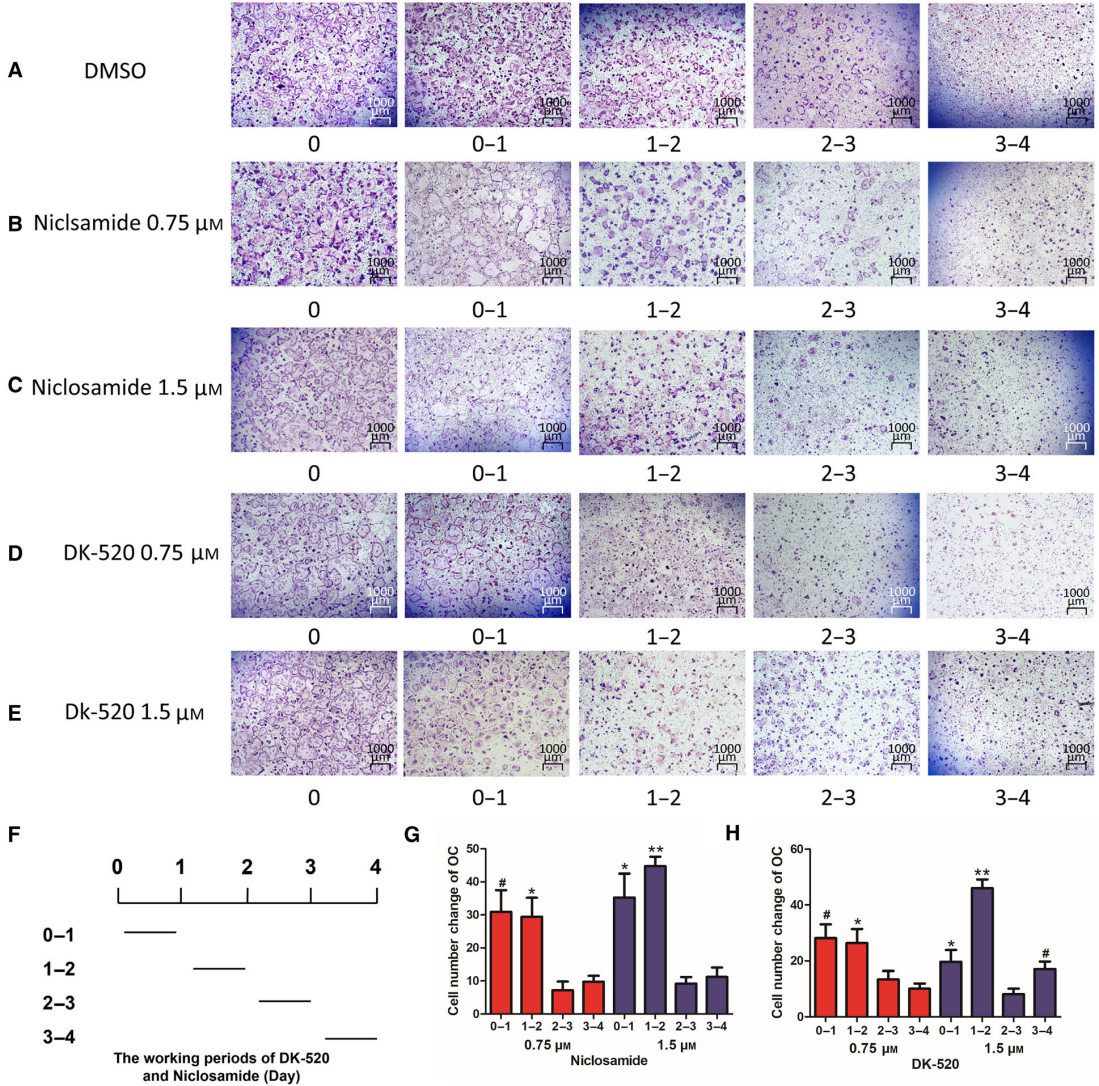
Both DK‐520 and Niclosamide inhibit osteoclast differentiation at the early stage of osteoclastogenesis. Following induction of osteoclast differentiation, cells were treated with vehicle or each compound at the indicated dose daily for 4 days. The morphology and TRAP expression in OCPs were assayed using enzymatic staining and counting the number of TRAP^+^ cells. (A–E) Both Niclosamide and DK‐520 altered the morphology and reduced TRAP expression in OCPs beginning on day 1. (F) Schematic illustration of treatments. (G,H) Both Niclosamide and DK‐520 decreased the number of TRAP^+^ cells. Data are representative images (magnification ×40) or expressed as the mean ± SD of each group from three separate experiments (*n* = 3). The data were statistically analyzed by Student’s *t*‐test. Scale bar: 1000 μm. ^*^ *P* < 0.05, ^**^ *P* < 0.001, ^#^ *P* < 0.01

**Fig. 4:**
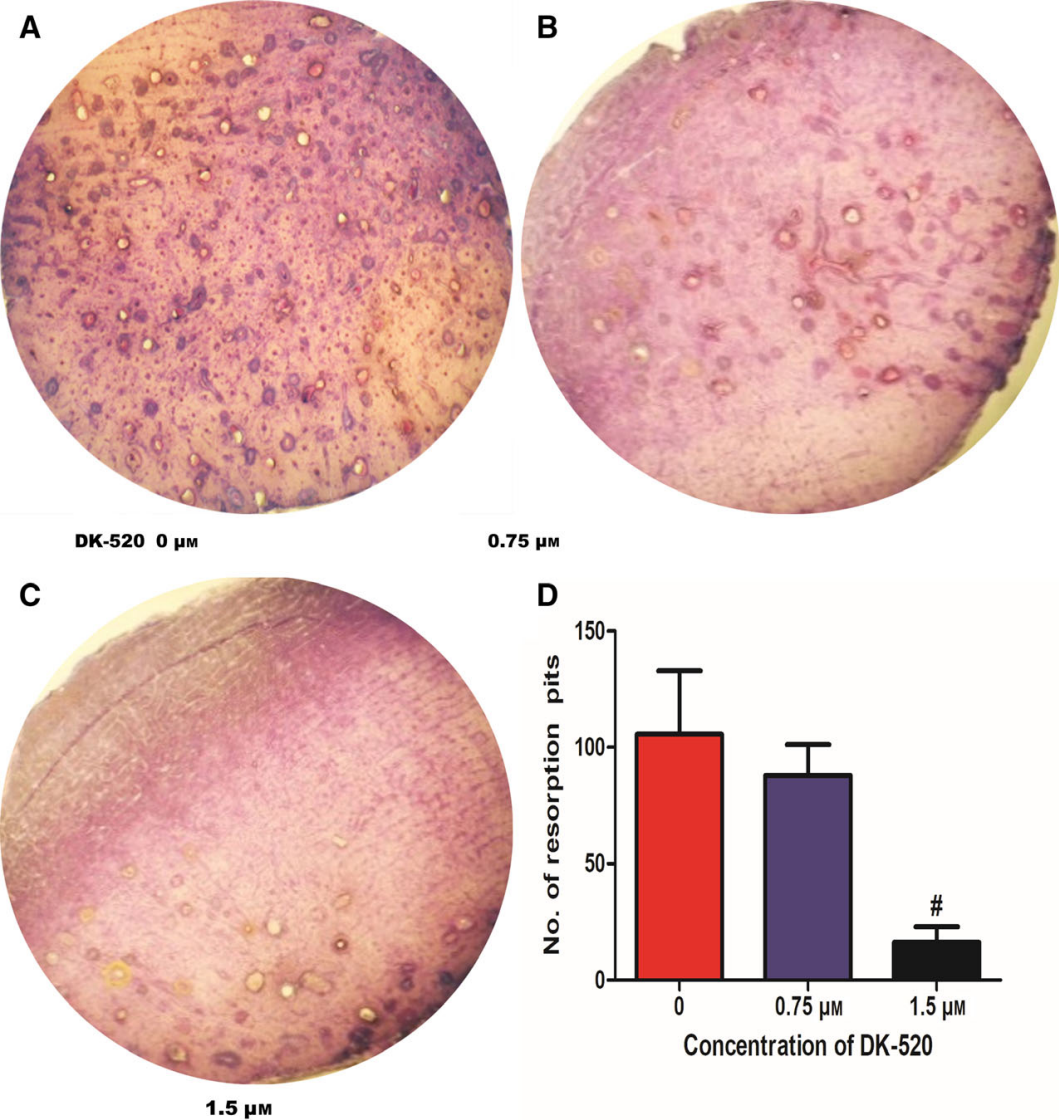
DK‐520 inhibits bone resorption in vitro with dose‐dependent manner. BMMs were seeded on bone slices and treated with M‐CSF (44 ng·mL^−1^) and RANKL (100 ng·mL^−1^), plus DMSO, 0.75 μm or 1.5 μm DK‐520 for 7 days. Cell culture medium was newly changed every 2 days. (A–C) Bone slices were stained with Toluidine blue, and resorbed pits were counted. (D) Data are expressed as the mean ± SD of each group from three separate experiments (*n* = 3). The data were statistically analyzed by Student’s *t*‐test, ^#^ *P* < 0.01.

Next, we explored whether Niclosamide and DK‐520 affected the formation and function of mature osteoclasts. Following induction of osteoclast maturation for 3 days, we found that both Niclosamide and DK‐520 at 1.5 μm, but not a lower dose, significantly reduced the number of osteoclasts, particularly following treatment with DK‐520 (Fig. 2F,G). Morphological analysis revealed that treatment with either Niclosamide or DK‐520 decreased the cell size and number of nuclei in the induced osteoclasts (Fig. 2C–E). These data suggest that both Niclosamide and DK‐520 can inhibit RANKL‐induced transdifferentiation of macrophages into mature osteoclasts *in vitro*. To determine the impact of each compound on RANKL activation, we observed osteoclast formation using histology (Fig. 3F). We found that treatment with either Niclosamide or DK‐520 at the tested doses reduced the cell body size, especially in the early stage of osteoclastogenesis (Fig. 3A–E), and decreased the numbers of TRAP^+^ cells compared with the DMSO group (Fig. 3G,H). The inhibitory effects of different doses of each compound tended to be dose‐dependent. To determine the role of DK‐520 to the function of mature osteoclasts, we generated osteoclasts in the dishes with cow bone slices, preincubated with RANKL and M‐CSF 2 days and added DK‐520 with various doses, cells were maintained in growth medium for 3 days and then removed cells which were on slices. As exhibited in Fig. 4A–D, the bone resorption pits were markedly reduced in DK‐520 groups, showing that it suppressed the function of mature osteoclasts and in a dose‐dependent manner. These data indicate that both Niclosamide and DK‐520 treatment can inhibit RANKL‐induced osteoclastogenesis and bone resorption *in vitro*.

### Niclosamide and DK‐520 inhibit PU.1 mRNA expression in the early stage of osteoclast differentiation

**Fig. 5:**
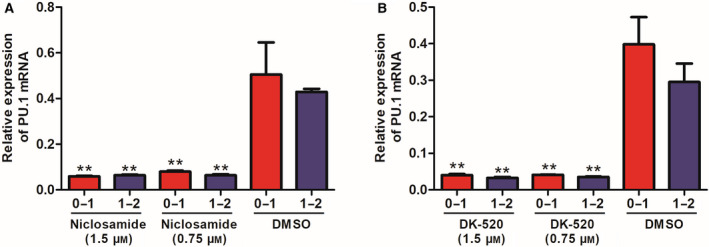
Both DK‐520 and Niclosamide inhibit the transcription factor PU.1 mRNA in OCPs. Following induction of osteoclast differentiation, the cells were treated with vehicle DMSO, DK‐520, or Niclosamide at the indicated doses daily for 2 consecutive days. The relative levels of PU.1 mRNA transcripts were measured using qRT‐PCR. Data are expressed as the mean ± SD of each group from three separate experiments. (A,B) Both DK‐520 and Niclosamide inhibited PU.1 mRNA transcript level in OCPs. Data are expressed as the mean ± SD of each group from three separate experiments (*n* = 3). The data were statistically analyzed by Student’s *t*‐test, ^**^ *P* < 0.001.

Because PU.1 is a critical transcription factor in the early stage of osteoclastogenesis, we further measured the relative levels of PU.1 mRNA transcripts daily after treatment with each compound using qRT‐PCR. Following treatment with either compound on day 1 or 2, the relative levels of PU.1 mRNA transcripts were dramatically reduced in the compound‐treated cells compared to the vehicle‐treated control cells (Fig. 5A,B). There was no significant difference in the inhibitory effects between these doses of compounds. The reduction in PU.1 mRNA transcripts by either compound demonstrates that both Niclosamide and DK‐520 can inhibit RANKL‐induced early transdifferentiation of macrophages into osteoclasts *in vitro*.

### Both Niclosamide and DK‐520 inhibit DC‐STAMP expression in differentiating osteoclasts

**Fig. 6:**
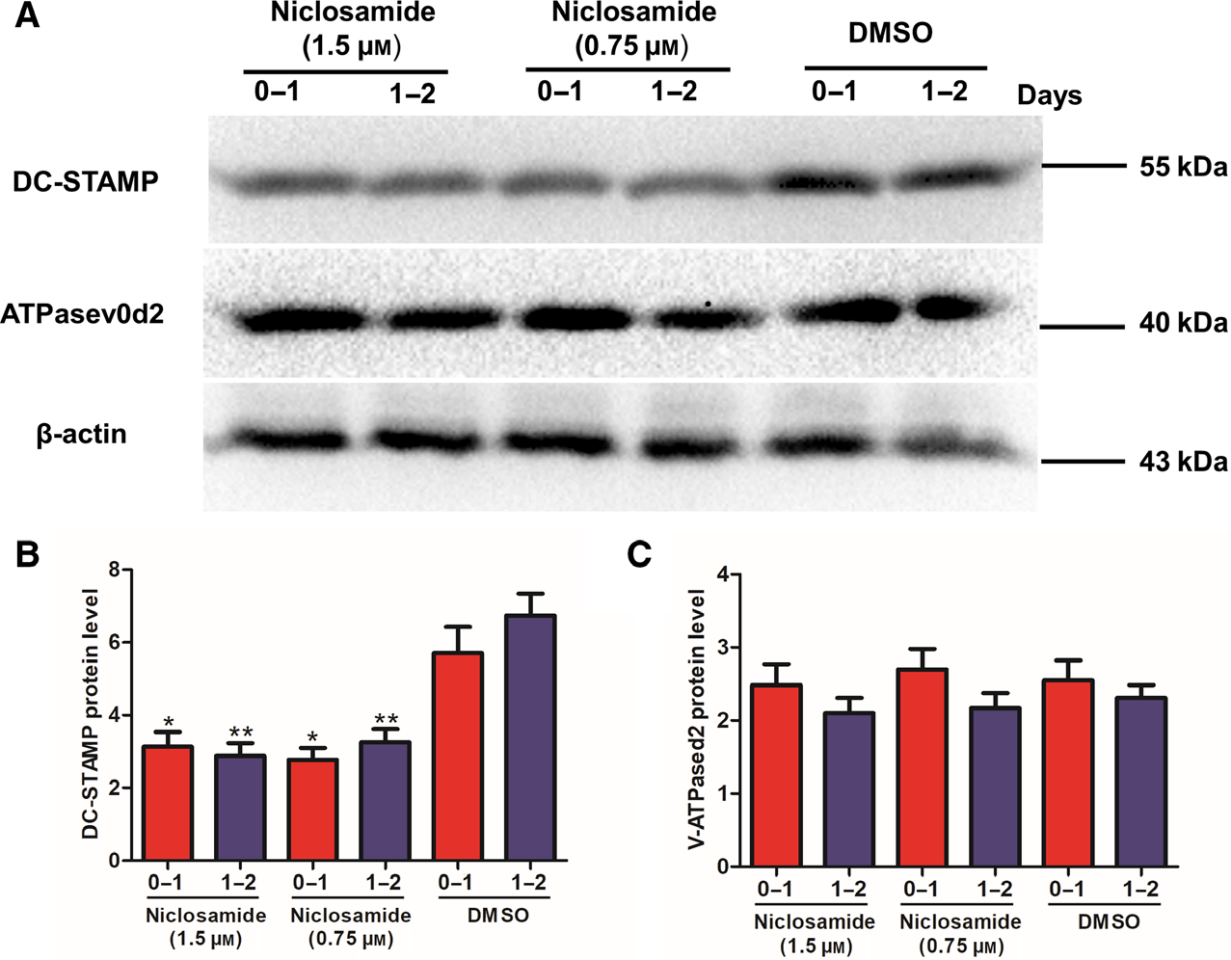
Niclosamide inhibits DC‐STAMP protein expression in OCPs. Following induction of osteoclast differentiation, cells were treated with vehicle, DMSO, or Niclosamide at the indicated doses daily for 2 consecutive days. The relative levels of DC‐STAMP and v‐ATPAse_0_d_2_ protein expression in each group of cells were measured using western blot analysis. Data are representative images or expressed as the mean ± SD of each group from three separate experiments. (A–C): Western blot analysis indicated that Niclosamide inhibited DC‐STAMP, but not v‐ATPAse_0_d_2_, expression in OCPs. Data are expressed as the mean ± SD of each group from three separate experiments (*n* = 3). The data were statistically analyzed by Student’s *t*‐test, ^*^ *P* < 0.05, ^**^ *P* < 0.001.

**Fig. 7:**
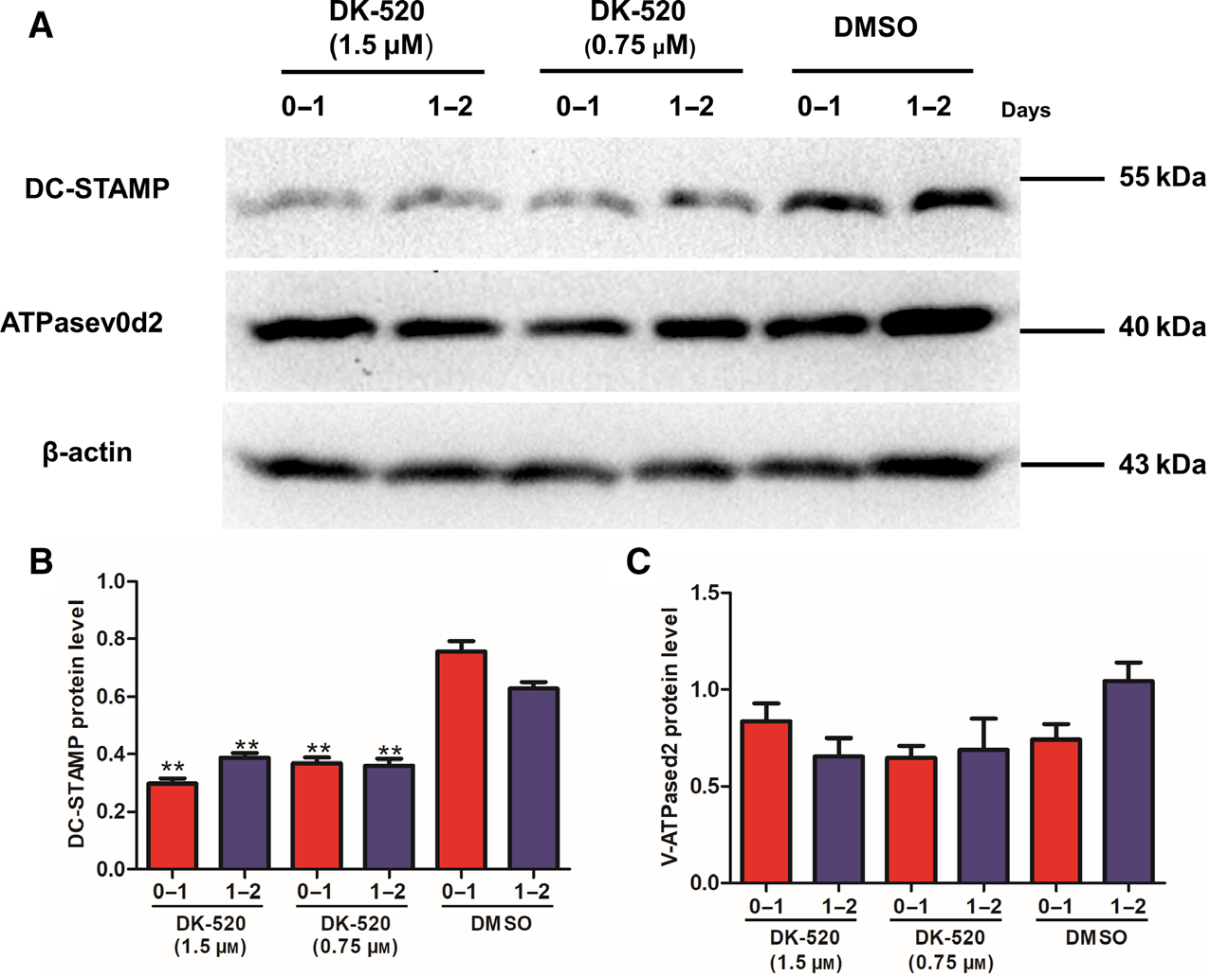
DK‐520 inhibits DC‐STAMP protein expression in OCPs. Following induction of osteoclast differentiation, cells were treated with vehicle, DMSO, or DK5‐520 at the indicated doses daily for 2 consecutive days. The relative levels of DC‐STAMP and v‐ATPAse_0_d_2_ protein expression in each group of cells were measured using western blot analysis. Data are representative images or expressed as the mean ± SD of each group from three separate experiments. (A–C): Western blot analysis indicated that DK‐520 inhibited DC‐STAMP, but not v‐ATPAse_0_d_2_, expression in OCPs_._ Data are expressed as the mean ± SD of each group from three separate experiments (*n* = 3). The data were statistically analyzed by Student’s *t*‐test, ^*^ *P* < 0.05, ^**^ *P* < 0.001.

Because DC‐STAMP and ATPaseV_0_d_2_ are crucial for the early process of osteoclastogenesis, we tested whether either Niclosamide or DK‐520 treatment modulated the expression of DC‐STAMP and ATPaseV_0_d_2_ in the RANKL‐treated cells using western blot analysis. The results indicated that treatment with either compound significantly decreased the relative levels of DC‐STAMP (Figs 6A,B and 7A,B), but not v‐ATPaseV_0_d_2_ (Figs 6A,C and 7A,C), expression in the differentiating cells compared to the control cells. Therefore, these data further support that both Niclosamide and DK‐520 can inhibit the early process of osteoclastogenesis *in vitro*.

### DK‐520 inhibits RANKL‐induced NFATc1 and C‐FOS expression

**Fig. 8:**
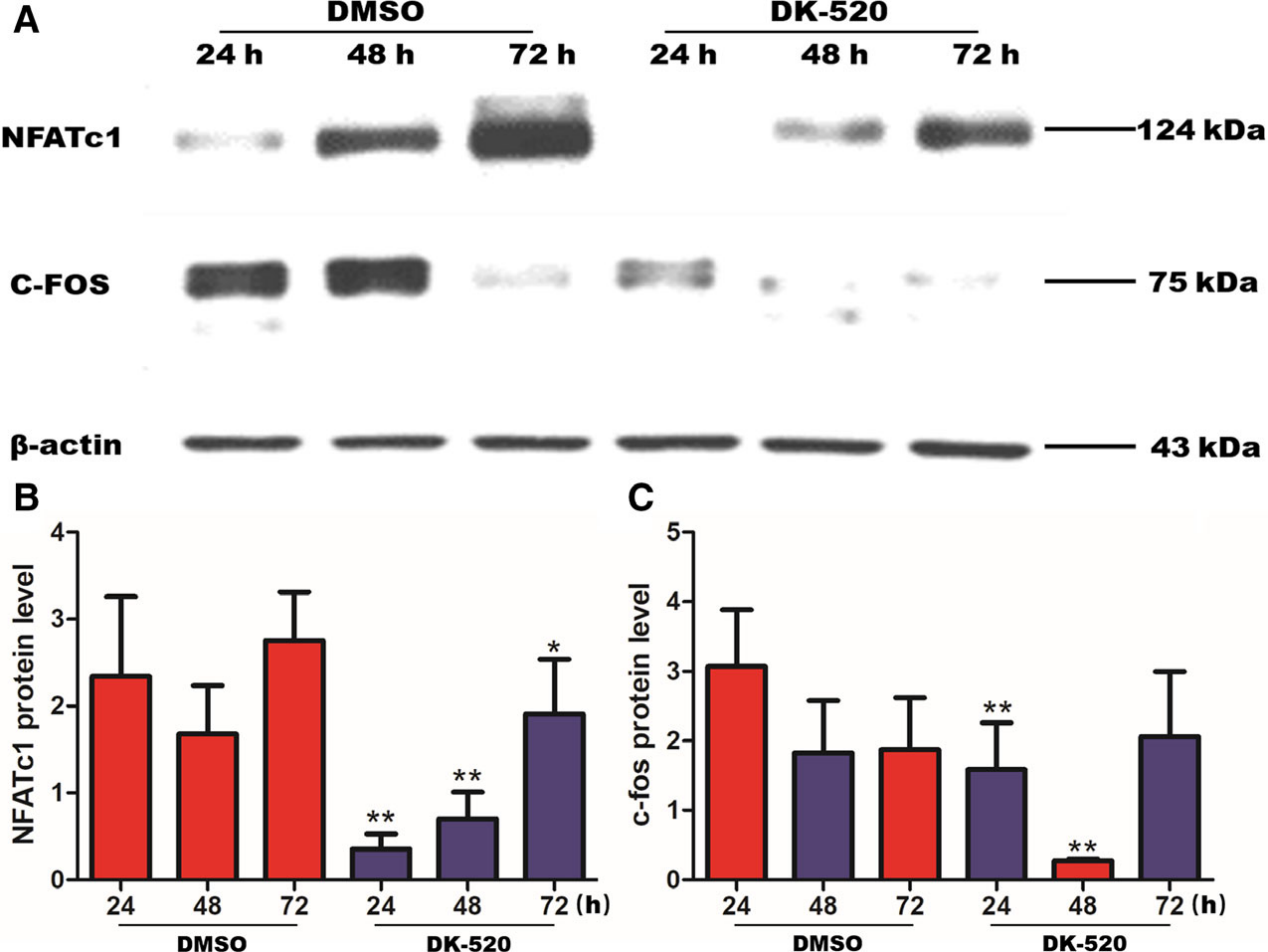
DK‐520 inhibits RANKL‐induced NFATc1 and C‐FOS expression. BMMs were seeded in 6‐well plate to culture with M‐CSF (44 ng·mL^−1^) and RANKL (100ng·mL^−1^) for 3 days to promote osteoclastogenesis. 1.5 μm DK‐520 was added at meanwhile, and after 24 h, 48 h, and 72 h, osteoclasts were lysed separately for western blot analysis. β‐actin was used as a loading control. Data are representative images or expressed as the mean ± SD of each group from three separate experiments. (A–C): Western blot analysis indicated that DK‐520 inhibited RANKL‐induced NFATc1 and C‐FOS expression. Data are expressed as the mean ± SD of each group from three separate experiments (*n* = 3). The data were statistically analyzed by Student’s *t*‐test, ^**^ *P* < 0.001.

We next explored the effect of DK‐520 on the expression of RANKL‐induced NFATc1 and C‐FOS in osteoclasts. Western blot revealed that DK‐520 inhibited NFATc1 and C‐FOS expression level in 24h, 48h, and 72h (Fig. 8A–C).

### DK‐520 inhibits osteoclastogenesis by affecting NF‐κB and MAPK signaling pathways

**Fig. 9:**
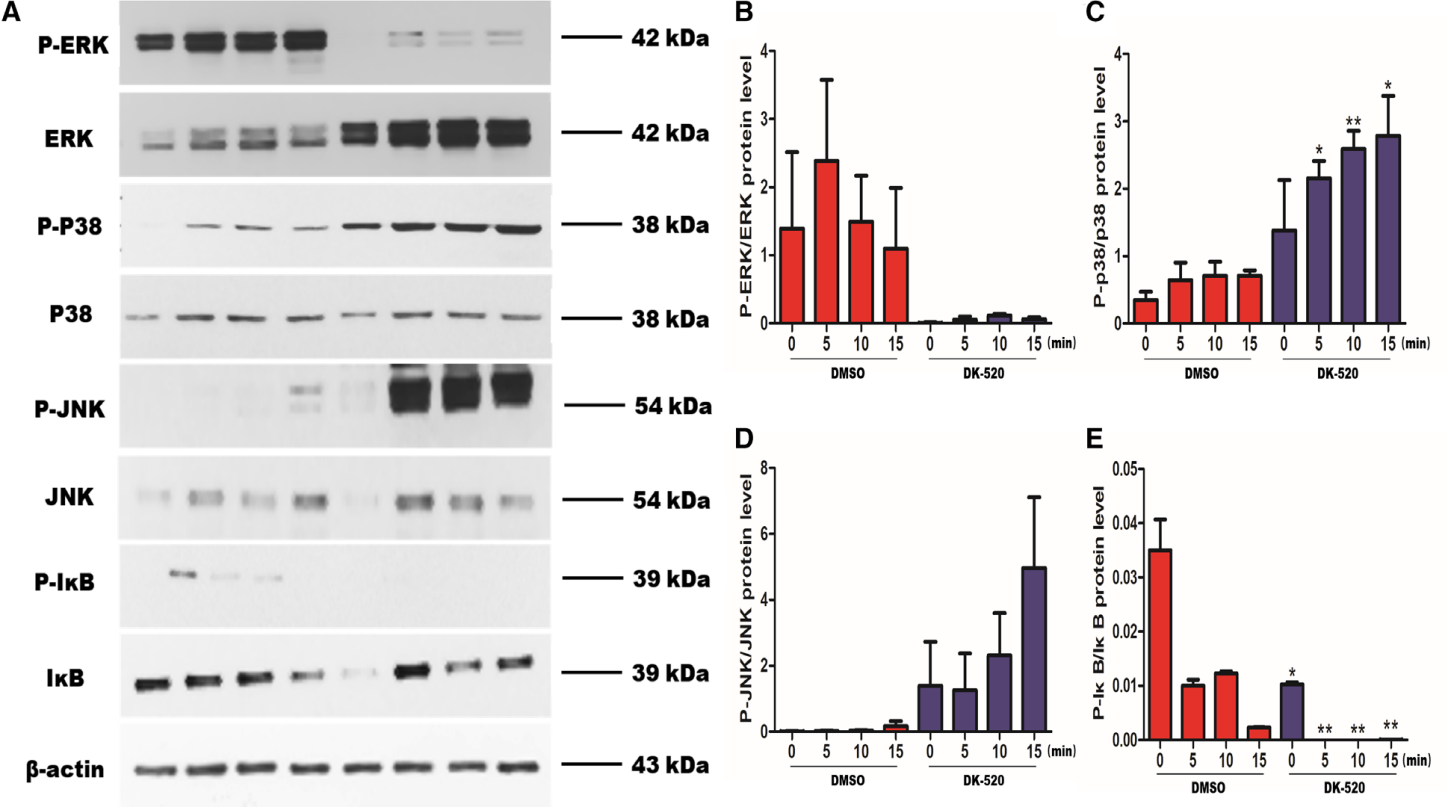
DK‐520 inhibits osteoclastogenesis by affecting NF‐κB and MAPK signaling pathways. BMMs were seeded in 6‐well plate to culture with M‐CSF (10%) for 2 days and changed medium without fetal bovine serum for 6h; then, 1.5 μm DK‐520 was added for 1 h, and cells were harvested without adding RANKL (0 min) or with adding 100 ng·mL^−1^ RANKL (5, 10, 15 min) separately for western blot analysis. The activation of NF‐κB and MAPK signaling pathways was assessed as phosphorylation of ERK, JNK, P38, and IκB using western blot analysis. BMMs cultured with adding DMSO instead of DK‐520 were used as control. Data are representative images or expressed as the mean ± SD of each group from three separate experiments. A: Western blot analysis indicated that DK‐520 inhibited the RANKL‐mediated p38 and the phosphorylation of ERK and IκB activation. (B–D) Histograms, respectively, show the protein levels of P‐ERK/ERK, P‐p38/p38, P‐JNK/JNK, and P‐IκB/IκB. Data are expressed as the mean ± SD of each group from three separate experiments (*n* = 3). The data were statistically analyzed by Student’s *t*‐test, ^*^ *P* < 0.05, ^**^ *P* < 0.001.

RANKL activates the NF‐κB and MAPK pathways. We then further assessed the effect of DK‐520 on the phosphorylation of ERK, JNK, P38, and IκB using western analysis. Our data showed that DK‐520 inhibited the RANKL‐mediated p38 and the phosphorylation of ERK and IκB activation (Fig. 9A–E).

## Discussion

In the current study, we assessed the effects and potential mechanism of Niclosamide and its derivative DK‐520 on RANKL‐induced osteoclast differentiation *in vitro*. We found that DK‐520, like Niclosamide, inhibited the early stage of osteoclast differentiation *in vitro*, supporting the findings that Niclosamide inhibits osteoclast differentiation and bone remodeling [17, 18]. More importantly, we found that DK‐520, like Niclosamide, significantly decreased both DC‐STAMP and PU.1, but not v‐ATPaseV_0_d_2_, expression in OCPs. Given that PU.1 and DC‐STAMP are crucial for early osteoclast differentiation, the significantly decreased expression of both molecules indicates that both Niclosamide and DK‐520 are able to inhibit the early process of M‐CSF‐ and RANKL‐dependent OCP fusion and differentiation *in vitro* [5, 20, 21]. Moreover, our results indicated that DK‐520 inhibits the RANKL‐induced osteoclastogenesis by attenuating the RANKL‐mediated p38 and the phosphorylation of ERK and IκB activation and suppressing the expression of c‐Fos and NFATc1 transcription factors. Our findings provide new insights into the pharmacological potential of DK‐520, as well as Niclosamide, for inhibiting osteoclast formation and subsequent bone resorption. Therefore, DK‐520 may be a promising new therapeutic for treating osteoclast‐related diseases [5].

PU.1 is a transcription factor in the ETS family that is crucial for the differentiation of macrophages, osteoclasts, and B cells [22, 23]. DC‐STAMP is a direct target of c‐Fos and NFATc1 and a master regulator of cell–cell fusion, and DC‐STAMP‐Tg mice display decreased bone mass and increased osteoclastogenesis, leading to the development of osteoporosis [24, 25]. We found that RANKL significantly increased PU.1 expression in OCPs, consistent with a previous report [26], and treatment with either DK‐520 or Niclosamide dramatically minimized PU.1 mRNA transcription and DC‐STAMP expression in OCPs. Given that PU.1 can induce NFATc1 expression, which promotes the expression of osteoclast‐specific genes and TRAP during osteoclastogenesis [27], and inhibition of OCP fusion reduces osteoclast activity and bone resorption and increases bone mass [24, 28], the decreased PU.1 and DC‐STAMP expression by either compound suggests that both compounds affect early OCP fusion during osteoclastogenesis. Since PU.1 deficiency or DC‐STAMP silencing completely abrogates cell–cell fusion and osteoclast formation during osteoclastogenesis [23, 29, 30, 31, 32, 33, 34, 35], PU.1 and DC‐STAMP may be new targets for inhibiting early osteoclastogenesis.

Interestingly, V‐ATPaseV_0_d_2_ is a component of the ATPase proton pump, which regulates osteoclast fusion and bone formation [36]. However, we observed that both DK‐520 and Niclosamide treatment failed to alter ATPaseV_0_d_2_ protein expression in OCPs. This suggests that neither compound affects ATPaseV_0_d_2_ expression and protein stability during OCP fusion. In future studies, we will investigate the pharmacological action of DK‐520 and Niclosamide in inhibiting osteoclastogenesis *in vivo*.

Osteoclasts are terminally differentiated myeloid cells. The formation of osteoclasts is a multistep process that is regulated by the NF‐κB and MAPK pathways leading to the induction and RANKL‐induced expression of c‐Fos and NFATc1 [37]. c‐Fos, a member of the AP family of transcription factors, is a critical switch involved in osteoclastogenesis, bone formation, and induction of downstream genes related to osteoclast differentiation [38]. RANKL‐RANK signaling leads to the robust induction of NFATc1 [37, 39]. A series of gene disruption studies has well‐documented the essential role of c‐Fos and NFTAC1 in bone homeostasis [39, 40]. Abrogation of these critical factors alleviates osteoclast formation and bone resorption [41, 42]. Early study reported that NFATc1^−/−^embryonic stem cells do not differentiate into osteoclasts [39]. Loss of c‐Fos shuttles redirects myeloid precursors toward macrophage commitment. Mice deficient in c‐Fos develop complete deficiency of osteoclasts with severe osteopetrosis [43]. Here, our results implied that DK‐520 drastically decreased the RANKL‐induced expression of c‐Fos and NFATc1 protein in osteoclast progenitors.

RANKL‐induced activation of c‐Fos and NFATC1 is downstream of the activation of the NF‐κB and MAPK pathways [44]. NF‐κB is a transcription factor and an inducible dimeric protein consisting of p65 and p50 subunits, which plays a central role in inflammation, autoimmune responses, differentiation, apoptosis, and cell proliferation [45, 46, 47]. Multiple previous studies have shown that NF‐κB plays a key role in osteoclastogenesis and its suppression affected NFATc1 expression [48, 49]. Genetic studies have shown that knockout of NF‐κB gene in mice results in severe osteopetrotic phenotype and failure of osteoclast formation [50]. Moreover, NF‐κB exists as inactive complex with inhibitory factor IκB which prevents nuclear translocation [48, 49]. Various inducers, including RANKL, can dissociate this complex and promote IκB degradation, presumably which liberates NF‐κB leading to its nuclear translocation and induces the gene transcription involved in osteoclast differentiation [51, 52]. In addition, the MAPK family includes JNK, ERK, and p38 which are relevant to osteoclast formation and play a crucial role in the modulation of osteoclast differentiation [53]. Abrogation of MAPK pathway severely alleviates osteoclast formation and bone resorption [53]. Inhibition of JNK abrogates RANKL‐stimulated osteoclastogenesis. Dominant‐negative ERK affects both osteoclast formation and survival, while inhibition of p38 activation severely blunts osteoclast differentiation but not osteoclast function [19, 54]. Therefore, NF‐κB and MAPK signaling pathways are a prerequisite for osteoclast differentiation and are critical to osteoclast function. In current study, we demonstrated that DK‐‐520 perturbed the RANKL‐induced phosphorylation and activation of p38, ERK, and IκB, whereas the expression of JNK was unaffected.

In summary, our results indicate that DK‐520, like Niclosamide, inhibits OCP fusion and osteoclast differentiation *in vitro* by significantly reducing PU.1 and DC‐STAMP expression. In addition, the inhibitory effect of DK‐520 on osteoclastogenesis is realized through impairment of the NF‐kB and MAPK signaling pathways. In addition, the inhibitory effect of DK‐520 on osteoclastogenesis is realized through impairment of the NF‐kB and MAPK signaling pathways. These findings provide new insights into the pharmacological action of DK‐520 and Niclosamide in inhibiting osteoclastogenesis and suggest that PU.1 and DC‐STAMP may be therapeutic targets. Our study also indicates that DK‐520, like Niclosamide, may be a promising new therapy for treating osteoclast‐related diseases.

## Conflict of interest

The authors declare no conflict of interest.

## Author contribution

YZ and XF conceived and designed the experiments. YJ and CC performed the experiments and prepared the manuscript. XH, ZS, JC, and QL analyzed the data.

## Data Availability

The data that support the findings of this study are available from the corresponding author upon reasonable request.
